# An overlooked phenomenon: complex interactions of potential error sources on the quality of bacterial de novo genome assemblies

**DOI:** 10.1186/s12864-023-09910-4

**Published:** 2024-01-09

**Authors:** Zoltán Rádai, Alex Váradi, Péter Takács, Nikoletta Andrea Nagy, Nicholas Schmitt, Eszter Prépost, Gábor Kardos, Levente Laczkó

**Affiliations:** 1 Institute of Metagenomics, University of Debrecen, Debrecen, Hungary; 2 Department of Dermatology, University Hospital Düsseldorf, Heinrich-Heine-University, Düsseldorf, Germany; 3 Department of Laboratory Medicine, Medical School, University of Pécs, Pécs, Hungary; 4 Department of Health Informatics, Institute of Health Sciences, Faculty of Health, University of Debrecen, Debrecen, Hungary; 5 Department of Evolutionary Zoology, ELKH-DE Behavioural Ecology Research Group, University of Debrecen, Debrecen, Hungary; 6 Department of Evolutionary Zoology and Human Biology, University of Debrecen, Debrecen, Hungary; 7 Department of Health Industry, University of Debrecen, Debrecen, Hungary; 8 Department of Gerontology, Faculty of Health Sciences, University of Debrecen, Debrecen, Hungary; 9 ELKH-DE Conservation Biology Research Group, Debrecen, Hungary

**Keywords:** Sequencing error, Sequencing depth, PCR duplicates, Optical duplicates, Bacterial genomes

## Abstract

**Background:**

Parameters adversely affecting the contiguity and accuracy of the assemblies from Illumina next-generation sequencing (NGS) are well described. However, past studies generally focused on their additive effects, overlooking their potential interactions possibly exacerbating one another’s effects in a multiplicative manner. To investigate whether or not they act interactively on de novo genome assembly quality, we simulated sequencing data for 13 bacterial reference genomes, with varying levels of error rate, sequencing depth, PCR and optical duplicate ratios.

**Results:**

We assessed the quality of assemblies from the simulated sequencing data with a number of contiguity and accuracy metrics, which we used to quantify both additive and multiplicative effects of the four parameters. We found that the tested parameters are engaged in complex interactions, exerting multiplicative, rather than additive, effects on assembly quality. Also, the ratio of non-repeated regions and GC% of the original genomes can shape how the four parameters affect assembly quality.

**Conclusions:**

We provide a framework for consideration in future studies using de novo genome assembly of bacterial genomes, e.g. in choosing the optimal sequencing depth, balancing between its positive effect on contiguity and negative effect on accuracy due to its interaction with error rate. Furthermore, the properties of the genomes to be sequenced also should be taken into account, as they might influence the effects of error sources themselves.

**Supplementary Information:**

The online version contains supplementary material available at 10.1186/s12864-023-09910-4.

## Background

With the substantial development and increasing availability of modern sequencing technologies there has been a rapid accumulation of whole genome data for prokaryotes. One of the most widely used technologies is the Illumina next-generation sequencing (NGS), which has become the mainstream method for high-quality sequence generation, and already has a number of high-throughput systems, such as MiSeq, HiSeq series, NextSeq, and the latest, NovaSeq. While the accuracy of NGS data has improved markedly in the past decade, no sequencing technology is without error, and most of the different technologies have their own typical error sources. Illumina NGS methods are no different, and are known to be characterized mainly by substitution errors (e.g. due to phasing error or between-cluster crosstalk) and the generation of optical duplicates [1, 2].

Although the so-called consensus adjustment incorporated in the final base calling may mitigate some of the substitution errors, a significant proportion of these still remain in the sequencing data [1]. Erroneous positions in the reads hinder de novo assembly of the genomes, mainly due to that one error may yield up to k erroneous k-mers, hence generating artifacts in the de Bruijn graph (e.g. parallel paths, chimeric connections, or unresolvable dead ends; [3]). Fortunately, some level of error correction is still possible by using tools specialized for that task, but the efficiency of these tools is not 100%, and depends on multiple additional factors, such as read length and sequencing depth [3, 4]. Downstream analyses are profoundly affected by the presence of erroneous positions in the sequencing data, as it will negatively influence the contiguity, completeness, and accuracy of the assembly, the identification of large-scale genome variations (from gene variants to genome structural polymorphism), or structural and functional annotation. Especially so when one does not have a reference genome on which to rely during assembly.

Beside erroneous base calls, PCR amplification prior to sequencing may also introduce additional errors, including occasional polymerase errors and the production of spurious read duplicates, the latter due to the non-uniform affinity of polymerases during PCR amplification (hence showing up in different clusters during sequencing). For Illumina sequencers, false duplicates can also originate from large clusters recognized as multiple clusters, or from re-clustering of library molecules, resulting in optical duplicates. PCR and optical duplicates are similar in the manner that they may bias downstream analyses by erroneously inflating estimated coverage and sequencing depth of certain genomic regions. A notable difference, however, is that PCR duplicates may contain errors originating from the polymerase’s activity, or from thermal damage to the DNA strands [5]. Nevertheless, the occurrence of spurious duplicates could lead to increased incidence of false positive findings in sequence variant calling, or biased heterogeneity indices across clones [6], although in some studies it was not found to significantly deteriorate the output and reliability of variant analyses [5]. Scaffolding can also be adversely impacted by duplicates, increasing both false positive and false negative result occurrence. More specifically, contigs may be incorrectly connected due to a higher incidence of connections (false positive), whereas contigs may be incorrectly disconnected due to a higher incidence of conflicting connections (false negative) [7]. Furthermore, PCR duplicates may also hinder genome assembly, particularly so in genomes characterized by extreme GC% values [8]. Notably, computational methods for marking and removing duplicates exist that are able to mitigate the duplication load in sequencing data [9]. Still, the presence of duplicates remains a non-trivial problem.

An additional factor that was shown to have substantial impact on de novo assembly from NGS data is sequencing depth (also referred to as read depth). A general rule-of-thumb among the molecular biologists and bioinformaticians appears to be that the higher the sequencing depth the better, with some consideration of balancing between “sequencing effort” *versus* “quality pay-off”. Previously it was shown that an intermediate sequencing depth can be regarded as optimal in de novo assemblies of relatively small genomes, such as of bacteria [10–12]. In these studies, no practically important adverse effects of high sequencing depth were ever identified, apart from being “unnecessary” or “wasteful”, based on its saturation-curve-shaped association with contiguity measures like N50 e.g. see [10, 13]. Notably, though, systematic errors may accumulate with high sequencing depths that could hinder assembly accuracy [14, 15]. However, past studies omit the analysis of potential interactions between multiple components that are otherwise considered to be deteriorative on assembly quality.

Furthermore, genomic properties (e.g. GC%, true genome size, ratio of unique genomic elements) may also affect de novo assembly quality [12, 16]. For example, it was shown that GC bias may affect sequencing depth itself, and can decrease assembly accuracy and increase fragmentation [17]. Also, as the genome size increases, the number and length of repeats typically increase, making it harder to resolve unique regions and properly reconstruct the genome. The size and complexity of a genome can therefore introduce difficulty to the assembly (the extent and nature of which may be dependent on the assembly algorithm) [18]. However, an overlooked question is whether or not these genomic properties may interact with error generating factors, potentially exacerbating their effects on the sequencing data, and consequently on de novo assembly.

A large number of genome assemblers utilize the de Bruijn graph algorithm, which is the most widespread mathematical representation of overlaps in short read sequencing data [19, 20]. By traversing the graph of overlapping short sequences (k-mers) it provides a way to reconstruct the original genome sequence from short overlapping subsequences. Because of its scalability and parallelization it has become a key component in many popular genome assembly algorithms and has significantly contributed to advancements in short read-based genome analysis. One assembler utilizing this approach is SPAdes (St. Petersburg genome assembler) a popular and reliable tool for de novo genome assembly [21]. Its user-friendliness (i.e. low number of mandatory input parameters, relatively short running time and high reliability) makes it a prominent example of a de Bruijn graph-based assembly software.

In summary, multiple factors might collude against the quality of sequencing data, potentially with substantial contributions to a lower quality genome assembly. While it is relatively well-known what factors may introduce errors, it is still largely unknown and overlooked whether their effects are additive or multiplicative, and in what ways may their interplay affect de novo assemblies. It is also not well studied how the genomic characteristics of the target genomes may influence not merely the assemblies, but the effects of the various sample parameters (e.g. sequencing depth, spurious duplicates). In our study we investigated the combined effects of important sample parameters on several measures of de novo assembly contiguity and accuracy. To that end, we simulated sequencing data for large numbers of genomes from multiple bacterial species to assess the additive and multiplicative effects of four crucially important sample parameters: sequencing depth, error rate, optical duplicate ratio, and PCR duplicate ratio. In addition, we assessed how the effects of these parameters are influenced by genome parameters, namely: genome size, ratio of unique genomic elements, and GC%. We utilized generalized linear models (GLMs) and generalized linear mixed-effects models (GLMMs) to quantitatively assess the associations between the described parameters and the quality of de novo genome assemblies, and a meta-analysis approach to pool effect sizes across bacteria, in order to identify general patterns in the effects of the assessed sample parameters on assembly quality.

## Results

Model outputs, estimated marginal trends, and pooled effect sizes are all available in the Supplementary Material. In addition, to aid interpretation of results of multiplicative models, we created an interactive online supplementary R-shiny app as well, for the visualization of model predictions. Both the static and the interactive Supplementary Material are available at https://github.com/DEpt-metagenom/Genome-error-simulations-supplementary.

### Assembly success

**Fig. 1:**
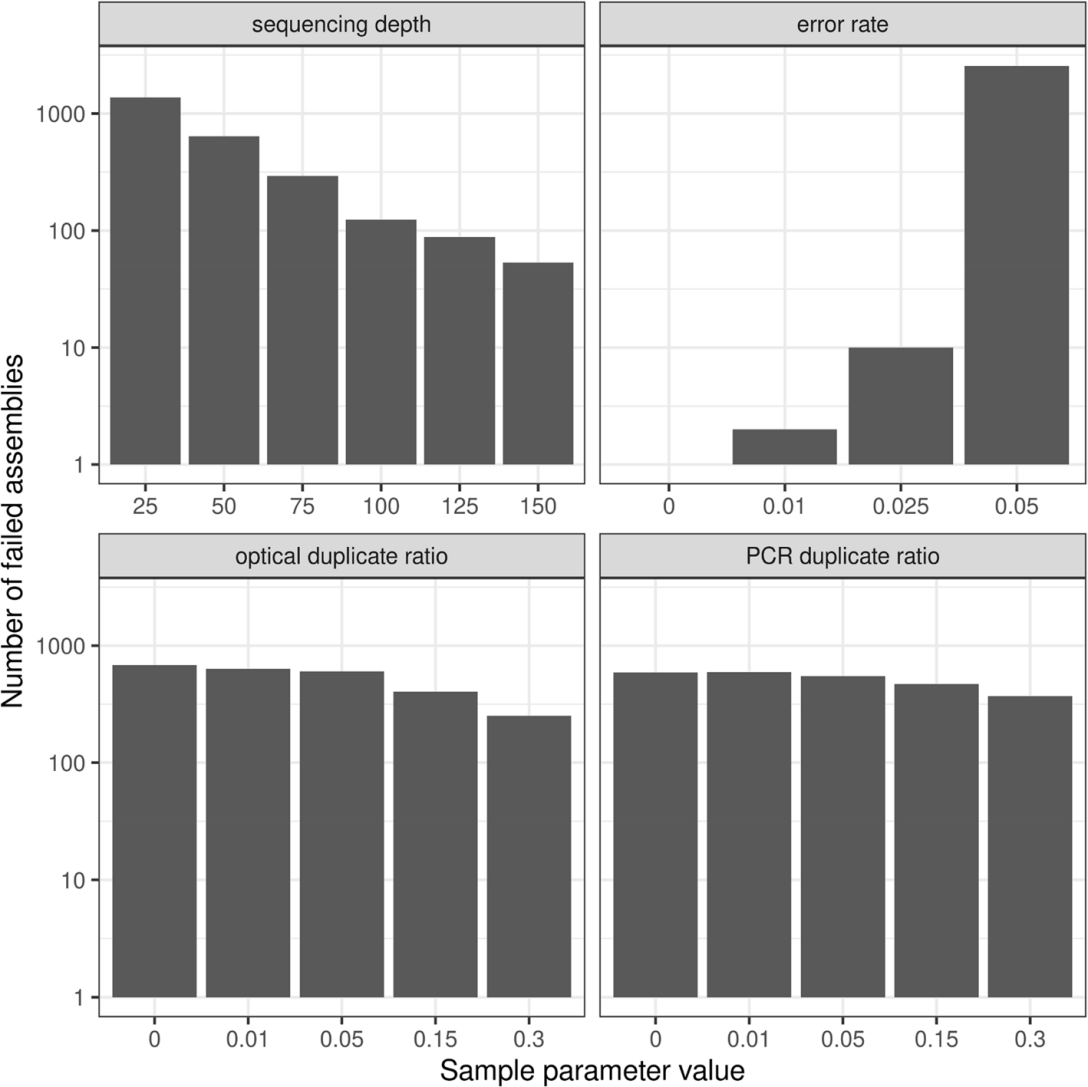
Number of failed assemblies in association with sample parameter values, separately shown for sequencing depth (top left), error rate (top right), optical (bottom left) and PCR duplicate ratio (bottom right). Vertical (y) axes are visualized in the log10 scale

Out of the 39,000 SPAdes de novo assembly attempts, 2569 (6.6% of the total) have failed (i.e. the assembly attempt did not produce contigs, because SPAdes stopped with an error, due to too large level of k-mer size distribution heterogeneity). Based on our binomial GLMM fitted on the presence of unsuccessful assemblies, these failures did not occur randomly: in fact, to some extent, all sample parameters contributed to their occurrence. Specifically, sequencing depth (ß = -3.799, SE = 0.094, *P* < 0.001), optical duplicate ratio (ß = -1.323, SE = 0.051, *P* < 0.001), and PCR duplicate ratio (ß = -0.681, SE = 0.051, *P* < 0.001) had a negative effect, whereas error rate had a strong positive effect (ß = 8.333, SE = 0.296, *P* < 0.001) (Fig. 1).

**Fig. 2:**
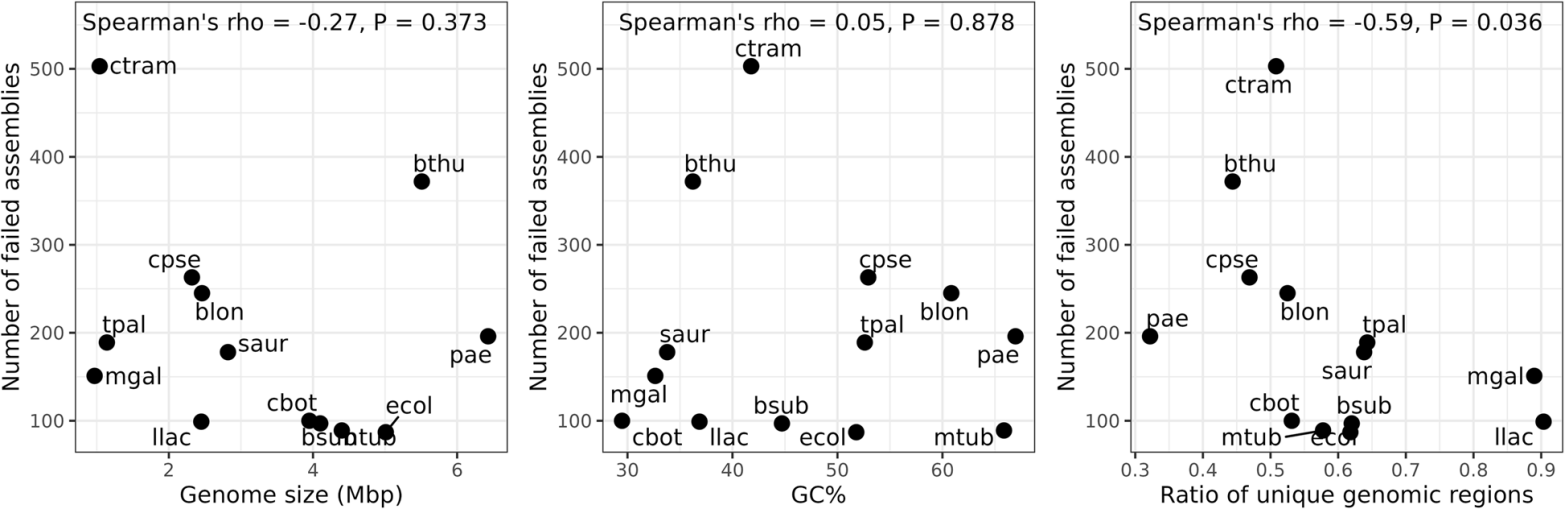
Associations between reference genome size (left), GC% (middle), and genome unique ratio (right) with number of failed assemblies. Sample names refer to the ID column of Table 1

The overwhelming majority of assembly failures occurred when error rate was highest (0.05; *n* = 2559). Reference genome parameters were largely uncorrelated with the number of failed assemblies (genome size: rho = -0.27, *P* = 0.373; GC%: rho = 0.05, *P* = 0.878; Fig. 2), although genome unique ratio showed suggestive negative correlation (rho = -0.59, *P* = 0.036).

### Quality metrics: additive models

**Fig. 3:**
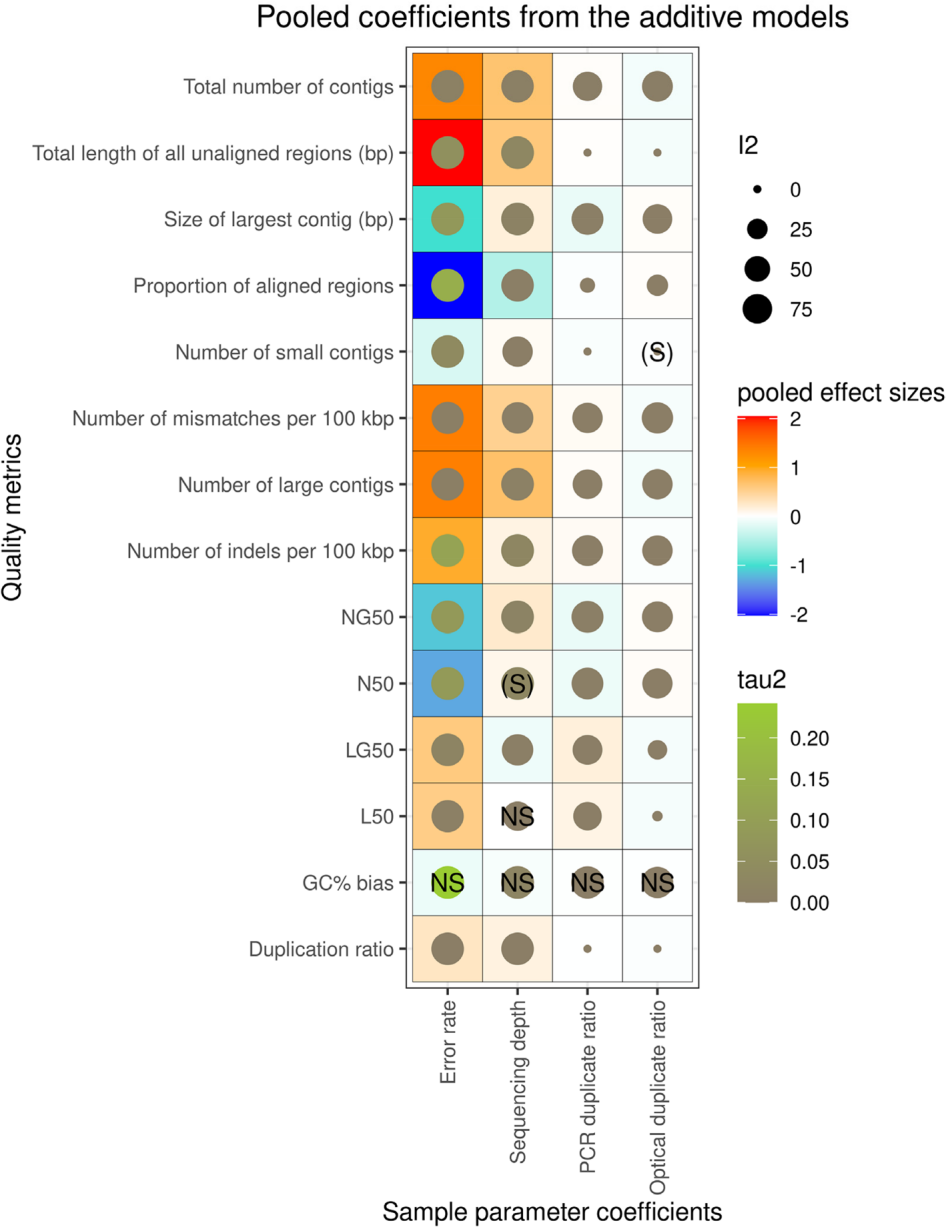
Visualization of the pooled effect sizes of the sample parameters on quality metrics, acquired from the additive GLMs. NS and (S) label non-significant (*P* > 0.05) and suggestive (0.003 < *P* < 0.05) association, whereas the absence of labels indicate significant (*P* < 0.003) effect. Blue and red colors in the tiles’ background indicate negative and positive pooled effect sizes, respectively. In addition, filled circles within tiles represent homogeneity measures from the meta-analytical models; specifically, their size is proportional to the meta-analytic models' between-reference-genome heterogeneity quantified by I^2^, whereas color represents the heterogeneity variance τ.^2^ (from grey to light green, from small to larger values, respectively)

Based on our meta-analyses, error rate tended to have the strongest effect on quality metrics, as shown by the generally largest effect sizes (see Fig. 3, and Supplementary Material).

We observed significant positive effect of error rate on the total number of contigs, number of large contigs, L50 and LG50, total length of all unaligned regions, duplication ratio, number of mismatches per 100 kbp, and the number of indels per 100 kbp. On the other hand, we found a significant negative effect on the number of small contigs, size of largest contig, N50 and NG50, as well as on the proportion of aligned regions. These results indicate that high error rate fragments larger sections of the genome assemblies, and abundant errors also make it harder to assemble small fragments, which in total lead to both lower contiguity and accuracy of assemblies. Error rate’s effect on the GC% bias was non-significant in the additive GLMs.

Sequencing depth tended to have relatively high effect sizes as well, although generally not as high as the error rate. It had a consistent positive effect on the total number of contigs, number of small contigs, number of large contigs, and the size of largest contig as well. Also, sequencing depth showed a suggestive positive association with N50 and significant positive association with NG50, whereas its pooled effect size on L50 did not significantly differ from zero, but showed significant negative effect on LG50. Furthermore, it showed a strong positive effect on the total length of all unaligned regions, duplication ratio, as well as the number of mismatches and indels per 100 kbp. Interestingly, it exhibited a relatively strong negative effect on the proportion of aligned regions. Overall, these results indicate that sequencing depth is positively associated with contiguity and some measures of assembly completeness, while negatively associated with accuracy and the final coverage of the original genome (as shown by the proportion of aligned regions). The effect of sequencing depth on GC% bias varied substantially across bacterial references, showing a non-significant pooled effect size.

Optical duplicate ratio generally had smaller effect sizes than error rate and sequencing depth. We found it to have significant positive effect on the size of largest contig, N50, NG50, and proportion of aligned regions, whereas it significantly negatively affected the total number of contigs, number of large contigs, L50, LG50, total length of all unaligned regions, duplication ratio, and the number of mismatches and indels per 100 kbp. We also observed a suggestive negative association with the number of small contigs. These effects, interestingly, seem to suggest optical duplicate ratio to have a small but effectively positive impact on de novo assembly quality via its positive effects on assembly contiguity and accuracy. In the case of optical duplicate ratio’s effect on the GC% bias the pooled effect size estimate did not significantly differ from zero.

In comparison to the optical duplicate ratio’s effects, a very similar yet inverse pattern emerged in the case of PCR duplicate ratio, with effect sizes of nearly the same magnitude, but opposite direction. Specifically, we found significantly negative effect sizes on the number of small contigs, size of largest contig, N50, NG50, and proportion of aligned regions. On the other hand, PCR duplicate ratio showed positive effects on the total number of contigs, number of large contigs, L50, LG50, total length of all unaligned regions, duplication ratio, and the number of mismatches and indels per 100 kbp. These results, overall, point towards small but consistently detrimental effects of PCR duplicate ratio on assembly contiguity, accuracy, and completeness as well. Its effect on the GC% bias was not significant.

### Quality metrics: multiplicative models

**Fig. 4:**
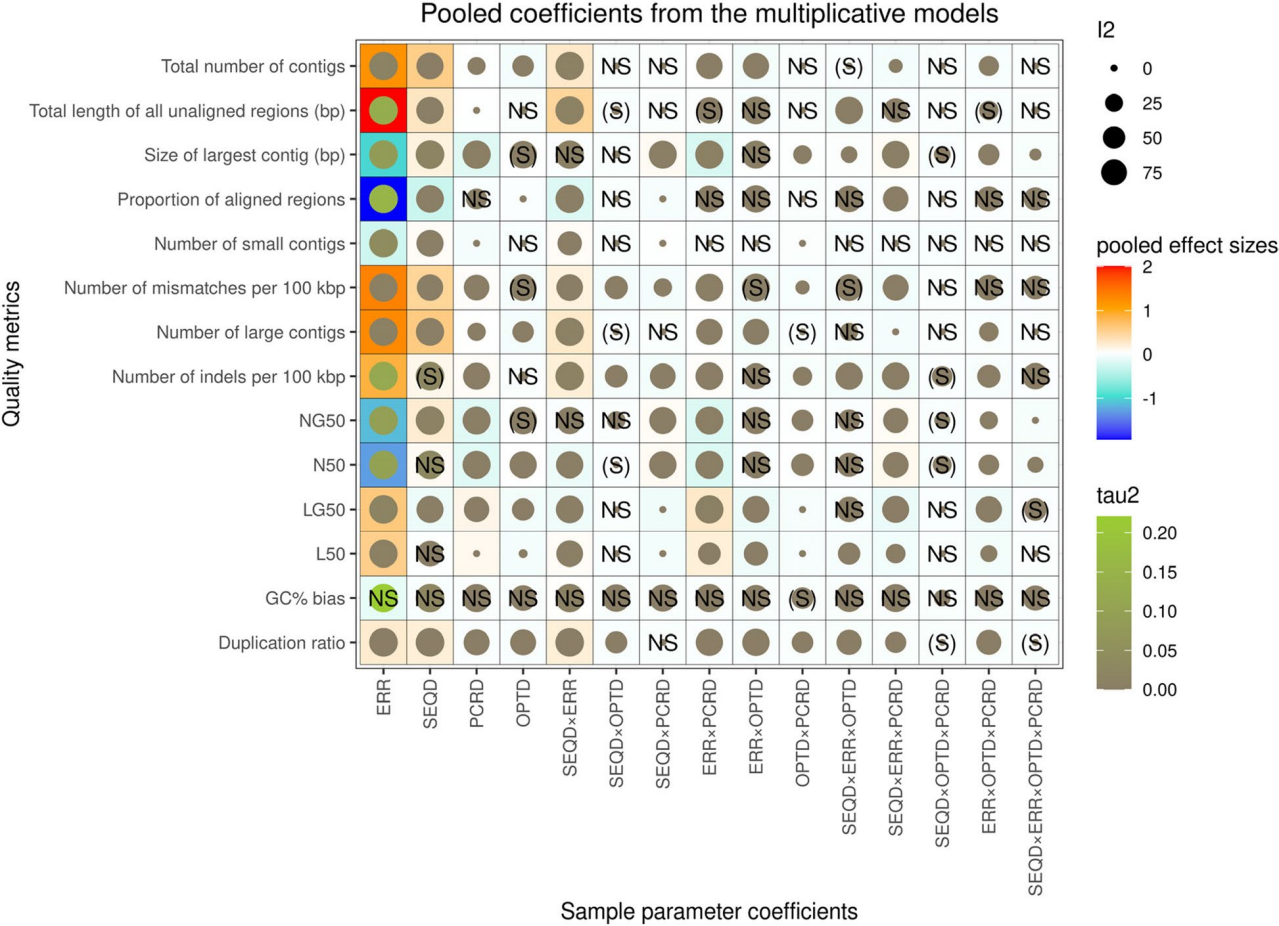
Visualization of the pooled effect sizes of the sample parameters on quality metrics, acquired from the multiplicative GLMs. Abbreviations for the sample parameters are: error rate (ERR), sequencing depth (SEQD), PCR duplicate ratio (PCRD), and optical duplicate rate (OPTD). Where sample parameters are connected with “ × ” represent the (pooled) interaction coefficients from the models. NS and (S) label non-significant (*P* > 0.05) and suggestive (0.003 < *P* < 0.05) association, whereas the absence of labels indicate significant (*P* < 0.003) effect. Blue and red colors in the tiles’ background indicate negative and positive pooled effect sizes, respectively. In addition, filled circles within tiles represent homogeneity measures from the meta-analytical models; specifically, their size is proportional to the meta-analytic models' between-reference-genome heterogeneity quantified by I^2^, whereas color represents the heterogeneity variance τ.^2^ (from grey to light green for small to larger values, respectively)

In a substantial number of models we found significant interactions between the sample parameters, from 2- to 4-way interactions. However, significant four-way interactions were rare (occurred only in 27 multiplicative GLMs out of 195), and the pooled four-way interaction term was found to be statistically significant only for the size of largest contig, N50 and NG50, and suggestively significant for LG50 and duplication ratio (see Fig. 4). Among the 3-way interactions the pooled interaction term was largely non-significant between sequencing depth, optical, and PCR duplicate ratios, with the exceptions of the size of largest contig, N50, NG50, duplication ratio, as well as the number of indels per 100 kbp, but even in these cases the level of significance was only suggestive (Fig. 4).

**Fig. 5:**
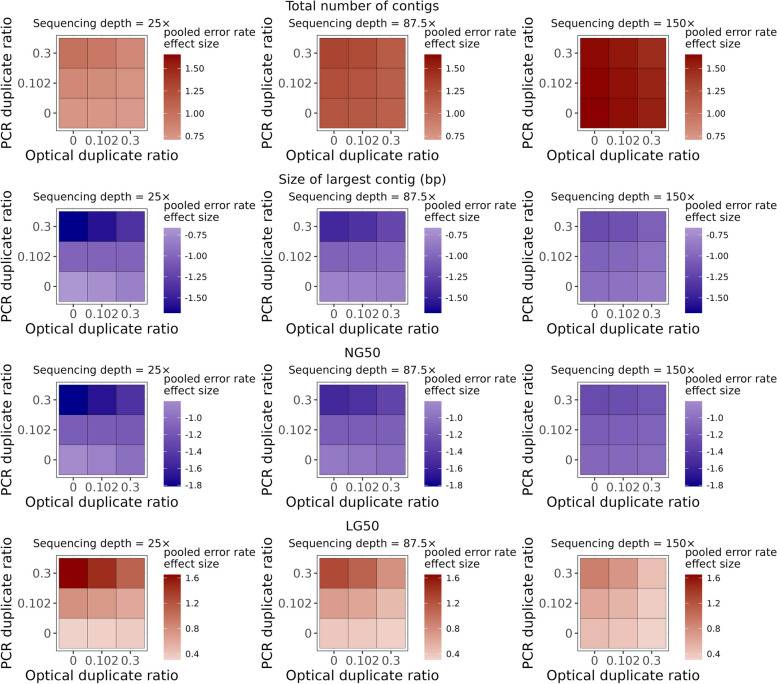
Visual representation of the pooled error rate effect sizes from the meta-analytic random-effects models in multiplicative GLMs on the total number of contigs (first row), size of largest contig (second row), NG50 (third row), and LG50 (fourth row). Error rate effect sizes were marginalized for different values of sequencing depth (25 × , 87.5 × , 150 × on the left, middle, and right columns, respectively), optical and PCR duplicate ratios (see on horizontal and vertical axes on the separate panels, respectively). Color intensity visualizes pooled error rate effect sizes, blue indicating negative, and red indicating positive effect. When present, the label (S) denotes suggestive (0.003 < *P* < 0.05), and NS denotes non-significant (*P* > 0.05) effects, whereas lack of labels indicate statistically significant (*P* < 0.003) effect of the pooled error rate estimate

**Fig. 6:**
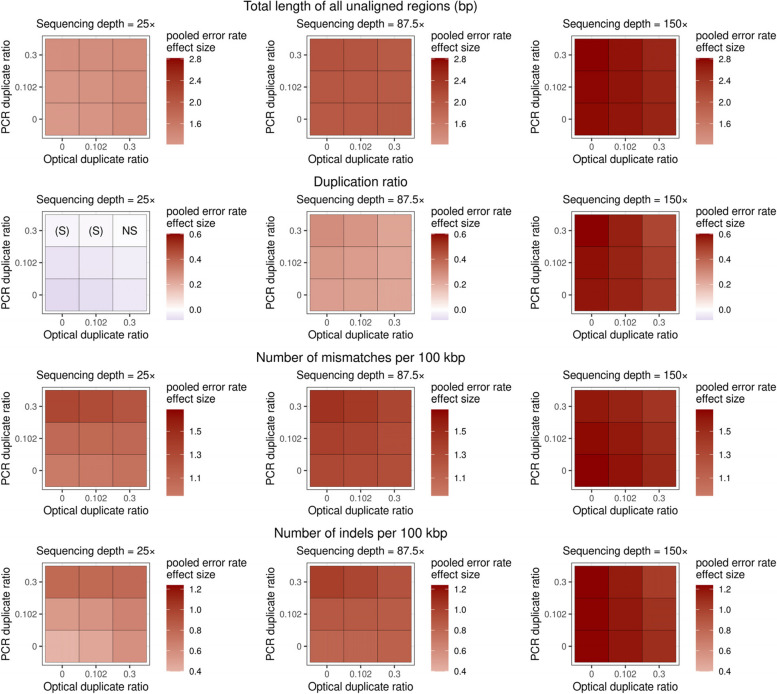
Visual representation of the pooled error rate effect sizes from the meta-analytic random-effects models in multiplicative GLMs on the total length of unaligned regions (first row), duplication ratio (second row) number of mismatches per 100 kbp (third row), and the number of indels per 100 kbp (fourth row). Error rate effect sizes were marginalized for different values of sequencing depth (25 × , 87.5 × , 150 × on the left, middle, and right columns, respectively), optical and PCR duplicate ratios (see on horizontal (x) and vertical (y) axes on the separate panels, respectively). Color intensity visualizes pooled error rate effect sizes, blue indicating negative, and red indicating positive effect. When present, the label (S) denotes suggestive (0.003 < *P* < 0.05), and NS denotes non-significant (*P* > 0.05) effects, whereas lack of labels indicate statistically significant (*P* < 0.003) effect of the pooled error rate estimate

In other quality measures 3-way interactions were quite often significant. The most diverse interactions were present, for example, in the case of the size of largest contig, N50, NG50, L50, LG50, and GC% bias (see Figs. 5 and 6). The most prominent 3-way interaction appeared to be the one between error rate, sequencing depth, and PCR duplicate ratio, being statistically significant for most of the tested quality metrics, with only three exceptions: total length of all unaligned regions, number of small contigs, and GC% bias. Based on the magnitude and direction of this interaction, error rate and PCR duplicate ratio often exacerbated each other’s effects, but sequencing depth can mitigate even their joint detrimental effects, by decreasing the strength of interaction between the two error-promoting effects.

Of the 2-way interactions, we observed the strongest interplay between sequencing depth and error rate, as shown by both the small number of non-significant pooled interaction terms (present only in models on NG50, the size of largest contig, and GC% bias), as well as the largest pooled interaction term estimates among 2-way interactions. Most notable were the cases of total length of all unaligned regions and the number of large contigs, the interaction between sequencing depth and error rate being positive, whereas it was negative in the case of the proportion of aligned regions. This is likely due to that an increased sequencing depth might help the recovery and assembly of the smaller sequence fragments. This also seems to be supported by the significant positive interaction between sequencing depth and error rate on the number of small contigs, meaning that higher sequencing depth mitigates the effect of error rate, and hence redeems small contigs which otherwise would not be possible to assemble due to the errors. Furthermore, higher sequencing depth can increase assembly contiguity by offsetting the fragmenting effect of error rate. On the other hand, the positive pooled interaction estimate between error rate and sequencing depth in the case of the total length of all unaligned regions, and the negative pooled estimate in the proportion of aligned regions suggest that higher sequencing depth enhanced the effect of error rate on the final size of the assembly, by enhancing the error rate’s effect via an increased occurrence of falsely duplicated segments (see duplication ratio quality metric). It should be noted, however, that its effect on contiguity was rather small, so it is unlikely to be the strongest drive of high contiguity when sequencing depth is at least 25, i.e. its effect is probably bears more weight when sequencing depth is low.

The second strongest 2-way interaction was apparent between error rate and PCR duplicate ratio; significant pooled interaction terms of notable magnitude were observed from models on the size of the largest contig (the interaction term being negative), N50 and NG50 (negative for both), and L50 and LG50 (with positive interaction terms for both). Pooled interaction terms were non-significant for the number of small contigs, proportion of aligned regions, and the GC% bias. This interaction effectively increased the detrimental impact of both parameters on assembly contiguity and accuracy, meaning that the negative impact of error rate and PCR duplicate ratio multiplicatively increment each other’s effects, instead of simply additively increasing inaccuracies and fragmentation.

### Quality metrics and reference genome parameters: additive models

Genome size had a suggestive enhancing influence on the error rate’s effect size on the number of indels per 100 kbp (i.e. the absolute value of the effect size increased with larger genome size), whereas it had (suggestively significant) reducing influences on the effect of error rate on the number of large contigs, and duplication ratio. This could indicate that the number of indels disproportionately increases due to error rate in larger genomes, although this sporadic influence of genome size seems to suggest very little (if any) role in shaping the assessed effects.

Higher unique ratio (i.e. the proportion of unique, non-repeated regions in the genome) was found to somewhat mitigate the detrimental effect of error rate on contiguity and accuracy, as it mitigated error rate’s (positive) effect on the total number of contigs. In more detail, it was found to have enhancing effect on a number of effect sizes, such as on the effect of sequencing depth on the total number of contigs, number of small contigs, number of large contigs, total length of all unaligned regions, duplication ratio, and mismatches per 100 kbp; on the effect of error rate on the number of small contigs, L50, total length of all unaligned regions, and duplication ratio; on the effect of PCR duplicate ratio on L50. Also, genome unique ratio was observed to exert a reducing influence on the effect of error rate on the total number of contigs and on the proportion of aligned regions; on the effect of optical duplicate ratio on the total number of contigs, number of large contigs, and proportion of aligned regions. Furthermore, the effect of PCR duplicate ratio on the GC% bias was diminishing in genomes of intermediate unique ratio, while effect sizes were significantly negative in low unique ratio genomes, and significantly positive in high unique ratio genomes. These seem to suggest that high unique ratio somewhat hinders error rate’s detrimental effect on contiguity and completeness, but still promotes error rate’s effects on inaccuracies and the production of small assembly fragments.

GC% of the reference genome had more nuanced influence on effect sizes than genome size and unique ratio. Our results seem to indicate that there is a substantial influence of GC% on how sequencing depth and error rate affect the assembly contiguity, as both of these sample parameters exert stronger effects in low-GC% genomes compared to high-GC% genomes. In most cases its influence was linear or closely linear (i.e. although the effect of the second degree polynomial was statistically significant, we did not observe hill- or U-shaped curvature upon visual inspection), but in some other cases we have found significant quadratic (or second degree polynomial) effects as well. Specifically: effect sizes were reduced by GC% in the effect of sequencing depth on the number of small contigs and on the size of largest contig. Similarly, effect sizes of error rate were reduced on the number of small contigs, L50, and the size of largest contig. In the case of GC% bias, effect sizes of PCR duplicate ratio, sequencing depth, and error rate tended to be significantly positive at low reference GC%, and significantly negative at high GC%. In contrast, we observed significant negative effect size of optical duplicate ratio on GC% bias at low reference GC%, but positive effect size at higher GC%. Additionally, effect sizes of sequencing depth on LG50 and on the number of indels tended to be largest at low and intermediate GC% values, respectively. Furthermore, the effect sizes of PCR duplicate ratio on both N50 and NG50 were largest at intermediate values of GC%. All-in-all, these results indicate that there is a substantial influence of GC% on how sequencing depth and error rate affect the assembly contiguity, as both of these sample parameters exert stronger effects in low-GC% genomes compared to high-GC% genomes.

### Quality metrics and reference genome parameters: multiplicative models

We assessed the influence of reference genome parameters on the pooled effect size of error rate, marginalized to different value combinations of sequencing depth, optical duplicate ratio, and PCR duplicate ratio (see Methods). This way we could infer on how genome parameters may shape the effect of error rate, at different value combinations of the other three sample parameters, i.e. whether or not the values of sample parameters affected how genome parameters shape error rates.

The enhancing influence of genome size in the case of error rate’s effect size on the number of indels was found to be strongest when sequencing depth was low, and at higher sequencing depths both PCR and optical duplicate ratio appeared to weaken the influence of genome size.

**Fig. 7:**
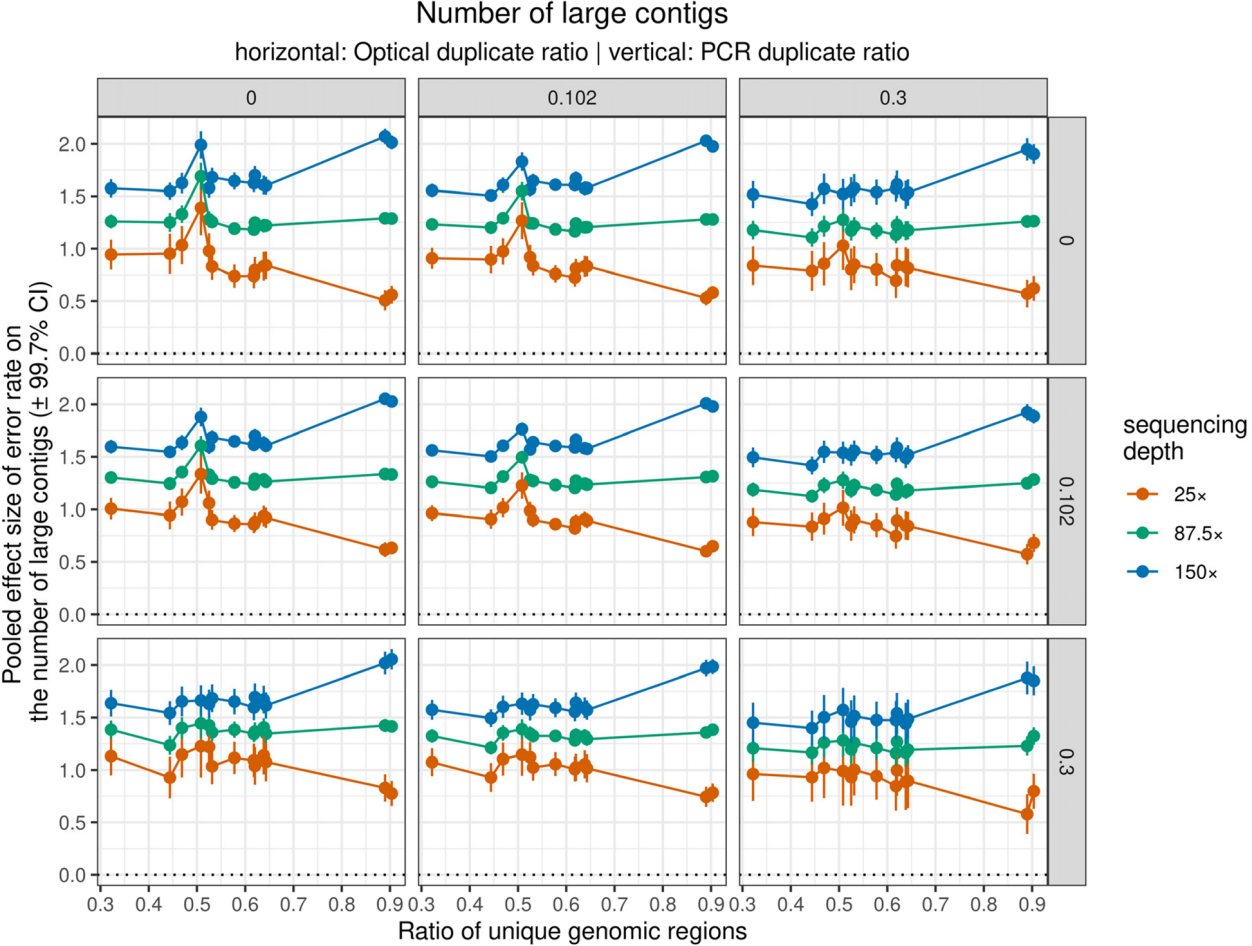
Influence of genome unique ratio (i.e. the ratio of non-repeated regions in the reference genome) on the error rate effect sizes on the number of large contigs. Different panels show the association at different value combinations of PCR (horizontal, i.e. rows) and optical (vertical, i.e. columns) duplicate ratio. Also red, green, and blue colors show effect sizes when sequencing depth was 25 × , 87.5 × , and 150 × , respectively

In the case of genome unique ratio, its influence on error rate effect size on the total number of contigs weakened at higher sequencing depth, and higher values of optical and PCR duplicate ratio also weakened its influence. The effect size of error rate on the number of large contigs was negatively associated with genome unique ratio when sequencing depth was low, but this association was positive when sequencing depth was high (Fig. 7).

For the effect of error rate on L50, when sequencing depth was low or intermediate, higher PCR duplicate ratios were linked with stronger influence of unique ratio, although higher optical duplicate ratio diminished this trend. At higher sequencing depths, unique ratio enhanced the effect of error rate on the total length of all unaligned regions more strongly. Also, at low and intermediate sequencing depths both optical and PCR duplicate ratios decreased this strengthening effect of sequencing depth, but at high sequencing depth (when the influence of unique ratio was strongest on the effect size of error rate on total length of unaligned regions), higher optical duplicate ratios somewhat decreased the strength of association. Additionally, error rate’s effect size on duplication ratio was enhanced by genome unique ratio at intermediate and high sequencing depths.

**Fig. 8:**
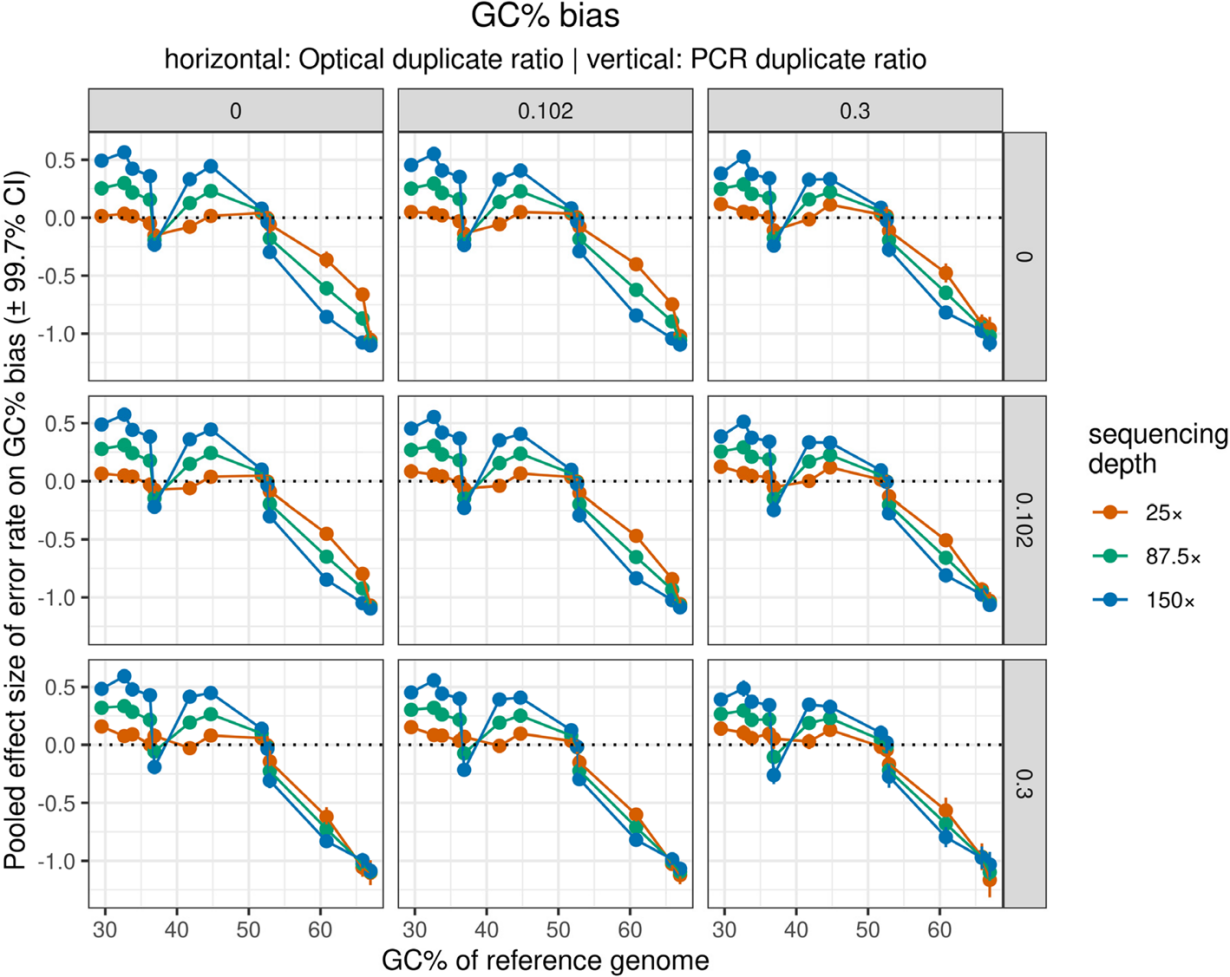
Influence of GC% of the reference genomes on the error rate effect sizes on GC% bias of the assemblies. Different panels show the association at different value combinations of PCR (horizontal, i.e. rows) and optical (vertical, i.e. columns) duplicate ratio. Also red, green, and blue colors show effect sizes when sequencing depth was 25 × , 87.5 × , and 150 × , respectively

The influence of reference genome GC% on the effect sizes of error rate was largely non-significant, with the peculiar exception of GC% bias, in which case original GC% maintained a strong negative influence on error rate effect sizes (Fig. 8). At low reference GC%, error rate effect sizes tended to be positive, whereas at high GC% effect sizes were negative. Furthermore, higher sequencing depth increased the steepness of this association, meaning that, when reference GC% was very low, or very high, the largest error rate effect sizes occurred under high sequencing depth. The result that the effect sizes of sample parameters on the GC% bias were also affected by the GC% of the reference genomes, draws a rather complex picture on the potential biases shaping assembly quality. Specifically, the original GC% of a genome seems to influence in what direction and to what extent sample parameters will distort the GC% of the assembly. Indeed, we saw that in low GC% genomes sequencing depth, error rate, and PCR duplicate ratio inflate GC-content of the assembly, whereas for high-GC% genomes these factors will decrease GC content.

## Discussion

### General assessment

Our findings underscore the importance of the background error rate, sequencing depth, and the presence of PCR and optical duplicates on the quality of de novo genome assemblies. More importantly, our results also highlight that these factors are not merely independent agents, but have significant interactions between them, meaning that their combined effects are substantially more complex than often assumed. We also observed that not only are the studied parameters engaged in a complex interplay, but their effects can be influenced by the original properties of the genomes we attempt to produce assemblies for. Awareness of the interplay between sample parameters, and the influence of genomic properties on them, may help us to gain a deeper understanding of how they shape assembly, and we can be more conscious and consistent in our attempts to produce assemblies of high quality.

Before discussing our results in detail, however, it is important to note the potential limitations of our approach. Firstly, by using only SPAdes for assembling genomes it remains an open question whether or not other assemblers differ in how the tested sample parameters affect assembly quality. In general, different short read assemblers may utilize the de Bruijn graph assembly algorithm differently [22], with the different approaches having their own specific strengths and weaknesses. It is outside of the scope of our current study to characterize assemblers, as it was already carried out by multiple studies (see [23] and references therein). Notably, though, in the future it might be worth extensively investigating how varying levels of sequencing depth, error rate, and presence of PCR and optical duplicates can affect assembler performances differently across the currently available software. It should also be mentioned that SPAdes attempts to automatically find the best k-mer lengths for analyzing the dataset and uses a multi-kmer strategy for assembly. A major difference between different short-read assemblers is how they handle k-mers and whether they use a universal k-mer size (e.g., Velvet or Minia) or a multi-kmer strategy (e.g., SPAdes or MEGAHIT). Although it may seem like a small difference, the assembly result can be drastically affected by how each software performs automatic k-mer selection or what k-mer lengths the user specifies for the assembler (see [23, 24]), since there may be a trade-off between contiguity and complexity when different k-mer sizes are used: shorter k-mers lead to a more complex de Bruijn graph with more nodes and edges, whereas longer k-mers simplify the graph but may result in fragmented assemblies with lower contiguity. Because optimality (i.e. a balance between complexity and contiguity) of k-mer size is the basis of all de Bruijn graph-based genome assembly software (although, to our knowledge, this has not been yet explicitly tested), we believe that the potential errors described above may have similar implications for assembly quality for different assembly software. As sequencing strategies and bioinformatics tools evolve, the optimal method for genome reconstruction may also change (e.g., [19]). The importance of different methods in the context of the proliferation of long-read sequencing may also be highlighted, which can lead to higher contiguity of assemblies. Although the above results are well applicable to the assembly of bacterial genomes with short reads, it might be worthwhile to investigate the impact of the specificities of sequencing methods on assemblies with long reads and hybrid methods (i.e., the simultaneous use of short and long reads), but this issue is beyond the scope of the current study. Despite the proliferation of new sequencing platforms—such as PacBio and Oxford Nanopore—Illumina sequencing is still widely used today, and we find it to be likely to remain the preferred platform for many genomics research projects because of its still-important advantages, such as higher read fidelity, parallelizability and the established infrastructure, protocols and expertise.

In our analyses we tested the associations between the sample parameters and assembly quality metrics using a generalized linear regression modeling approach. The assessed quality metrics varied substantially in their value distributions (e.g. some of them being continuous, others discrete) and distribution shapes (e.g. heavily right or left skewed), making it difficult to identify adequate model families. While transforming quality metrics to the [0,1] standard limit enabled us to use the Beta model family, which robustly handles more exotic value distributions, it has to be noted that using a continuous distribution model family on discrete data might ignore some of the inherent properties of the modeled value distributions (e.g. number of contigs, or N50). Therefore in our parametric approach we might have traded off flexibility and interpretability with some level of accuracy. Consequently, parameter estimates and model predictions should be interpreted with caution, and be taken as broad guidelines towards understanding the modeled associations, rather than as accurate estimations. Nevertheless, we think that our results provide a robust and intriguing addition to our general understanding of how the studied sample parameters shape de novo genome assembly quality.

Notably, throughout the assembly of simulated genome sequencing data in some cases no assemblies were produced due to the high error rates. Therefore, even though our study had a complete block design in terms of sample parameter value combinations, in our analyses a number of observations (*n* = 2569, 6.6% of all assemblies) was missing-not-at-random (MNAR). Consequently, we had more complete data with lower error rates. However, given that high error rates generally were associated with low assembly quality, and that the overwhelming majority (*n* = 2559, 99% of genomes with 0.05 error rate) of assembly failures occurred at the highest error rate (0.05), it is likely that our effect size estimates were actually somewhat underestimated by the fitted models due to this case of MNAR.

In our study, we relied mainly on contiguity metrics to assess the effect of error sources. With the rise of third-generation sequencing (TGS), such as PacBio and Oxford Nanopore Technologies, some of the presented error sources might not be limiting factors anymore since both technologies offer PCR-free methods for the sequencing library construction and do not produce clusters of reads prior to sequencing. The development of new sequencing technologies can make the application of some contiguity statistics (such as the N50) superfluous and draw the focus on the assessment of completeness, e.g. by using BUSCO [25]. In contemporary research the Illumina technology still remains a widespread standard of genome sequencing to obtain draft genome sequences, and we argue that the effect of the error sources presented above should be understood and accounted for correctly.

Finally, it should also be noted that our current approach was purely based on simulated sequencing data. Therefore one limitation is the lack of ability to experimentally confirm the interplay of the assessed sample parameters on the average assembly quality. In the future it might be worth to assess the estimated error rates and duplicate ratios, as well as the utilized sequencing depth, in association of the quality of the final assemblies on real world data.

### Effects of sample parameters on quality metrics

Unsurprisingly, higher error rates increased fragmentation of the assemblies, and decreased total alignment lengths, i.e. had a strong negative effect on both contiguity and accuracy of the assemblies. Interestingly, sequencing depth was also found to increase the number of contigs, but likely not due to increased fragmentation, but due to the increased capacity of the assembler to build contigs based on the higher availability of sequencing data. Indeed, the size of the largest contig was positively affected by sequencing depth. Also, both the number of small and large contigs increased with higher sequencing depth, whereas error rate had a positive effect only on large contigs, but a negative effect on small contigs. So while increased error rates fragment and spoil assemblies, an increased sequencing depth might help the recovery and assembly of the smaller fragments.

Sequencing depth was negatively associated with accuracy (see number of mismatches and indels, duplication rate). This could be explained by the interaction between error rate and sequencing depth, in which higher sequencing depth appears to amplify the effect of error rate, by providing stronger support for erroneous base positions during the assembly. Overall, the results discussed above seem to indicate that higher sequencing depth may help increase contiguity, but it also may exacerbate the effects of error rate leading to lower accuracy. For instance, their interaction likely leads to the increased occurrence of falsely duplicated contigs (as seen in their effects on duplication ratio in the assemblies). While the result that substantially increased sequencing depth may exacerbate effects of sequencing errors and lead to increased misassemblies and duplicated sequences, this is likely to originate from the artificially increased confidence in heterogeneous segments, leading to branches in the assembly graph that do not exist in the original genome. This way the erroneous contigs will be assembled as the slightly different duplicate of the original sequence (as seen by the increased duplication ratios in the assemblies by higher sequencing depths, and the positive interaction of error rate and sequencing depth on duplication ratio).

PCR duplications contributed to lower contiguity and accuracy in the assemblies. It also increased the duplication ratio, i.e. was causing the assemblies to have an increased number of contigs which covered the same regions of the reference genome. This effect likely stems from the fact that PCR duplicates are not perfect copies of the original reads, but are burdened by PCR errors [5]. These errors then play a substantial role in generating false heterogeneity in similar regions by introducing erroneous base positions, which are difficult to resolve during assembly. This seems to be in line not only with the similar effects of PCR duplicate ratio and error rate on quality metrics (in direction if not in magnitude), but also with the observation that these sample parameters consistently magnified each others’ effects (as shown by their 2-way interaction estimates). PCR amplification has long been an important step of sample preparation, ensuring the adequate amount of DNA fragments for sequencing. However, recently adapted techniques offer the possibility for PCR-free library preparation, which might help to alleviate the problem of PCR-related errors, such as duplicates and erroneous fragments [12, 26].

Optical duplicate ratio’s opposite effects to PCR duplicate ratio likely can be explained by it inflating confidence of De Bruijn graphs of some regions during assembly. Certainly, optical duplicates represent perfect clones of read data and may help in the resolution of faulty segments. This could also contribute to a mitigating effect on assembly errors such as mismatches, indels, or duplication ratio, suggesting that in de novo assembly, optical duplicates may help weaken the effects of other error introducing factors. In fact, the effect size of error rate was often dampened to some extent at higher optical duplicate ratios (see Figs. 5 and 6). Notably, though, the effect sizes belonging to optical duplicate ratio were about an order of magnitude smaller than that of sequencing depth or error rate, indicating that even at considerable rates of optical duplications, these beneficial effects may manifest in a quite restrained manner.

### Influence of reference genome properties on sample parameter effects

In general, genome size did not show substantial influence over the effect sizes of sample parameters, with some exceptions on error rate, in which cases larger genome sizes appeared to reduce error rate’s effect of fragmentation and inflating duplication ratio in the de novo assembly, although in larger genomes error rate was found to introduce more indels per 100 kbp in the assembly.

As for unique ratio, our results can be put into context by considering that with the increased proportion of repeated regions, low genome complexity was described to be associated with increased fragmentation of the assemblies [27, 28]. This is chiefly due to long tandem repeats, which cannot be adequately covered by the used insert size and/or read lengths, making them difficult (or even impossible) to resolve with de Bruijn graph assemblers. Notably, genomes with low unique ratio are likely also more prone to fragmentation and low assembly accuracy due to a non-zero error rate, because erroneous positions hinder the placement of the given genomic section into any contig, even if the length of the repeat-containing region is not very large.

Although in the past it was shown that GC% can affect assembly contiguity through its effect on sequencing depth [17], our results highlight a yet understudied phenomenon of how de novo assembly quality may be further affected, even when sequencing depth is relatively uniform across the genome. In addition, the effect sizes of sample parameters on the GC% bias were also affected by the GC% of the reference genomes, drawing a rather complex picture on the potential biases shaping assembly quality. To put more simply: the original GC% of a genome seems to influence in what direction and to what extent sample parameters will distort the GC% of the assembly. Indeed, we saw that in low GC% genomes sequencing depth, error rate, and PCR duplicate ratio inflate GC-content of the assembly, whereas for high-GC% genomes these factors will decrease GC content. In addition, as seen from the results of the multiplicative models, higher sequencing depth is not merely additive in its effect, but it enhances the effect of error rate, i.e. increased sequencing depth exacerbates the error rate's effect in low-GC% genomes. In the past it has been shown that substantially low and high GC% might impair sequencing depth and assembly quality [17, 29]. Although it is widely accepted that the assembly of repetitive genomes (that in many cases can be characterized by extreme GC%) require a higher effort both in terms of sequencing and assembly, to our best knowledge it was not known until now that even at relatively homogeneous sequencing depth very low or high GC% of the analyzed genome plays a complex role shaping the contiguity and GC% of de novo assemblies as well. While in our study we were able to highlight this phenomenon, it remains difficult to find an explanation. One plausible explanation appears to be that at decreased genomic complexity (manifesting in a reduced variability in the contributing bases in the sequence, hence e.g. leading to increased incidence of repeated segments) hinders the assembler’s capacity to reconcile ambiguous parts, even when variation in sequencing depth is small.

## Conclusions

Different sources of error were found to act not only additively, but they also exhibit substantial interplay, potentially exacerbating each other’s effects on the contiguity and accuracy of de novo genome assemblies. Such complex interactions may be further influenced by characteristics of the sequenced genome (mainly the proportion of unique, non-repeated regions in the genome, and GC%). In light of our results we recommend careful consideration of choosing the optimal sequencing depth, balancing between its positive effect on contiguity and negative effect on accuracy due to its interaction with error rate. Also, utilization of PCR-free library preparation may help not only against duplicates and erroneous reads, but could also mitigate the negative effect of error rate on assembly contiguity and accuracy, due to the absence of their interaction. Admittedly, it remains non-trivial to acquire error rates, and PCR and optical duplicate ratios for real-life data. Also, data on genomic properties of yet uncharacterized bacterial strains or species may not be available either. Fortunately still, a number of approaches or computational tools are available, which may provide ways of estimating some corresponding parameters (see [30–32] and references therein). For instance, error rate assessment and filtering can be done with fastp [33], a widely used robust tool, while sambamba [34] and picard (https://broadinstitute.github.io/picard/) can provide ways for duplicate identification. Such bioinformatical tools and prior experience with specific library preparation and sequencing protocols should enable researchers to identify sequencing depth ranges that are expected to provide optimal de novo bacterial genome assemblies.

## Methods

### Read simulation and assembly

To simulate raw sequencing data for bacterial genomes, we selected 13 bacterial reference genomes which covered a reasonably wide and heterogeneous range of combinations for genome size, GC%, and proportion of non-repeated regions (hereafter referred to as unique ratio; see Table 1), while also keeping in mind the potential relevance in applied and basic research of the chosen bacteria. In our statistical analyses we used the ratio of unique genomic regions as the total length of repeat sequences identified by Red [35], divided by the genome size.

**Table 1:** Summary for the selected bacterial reference genomes

Bacteria	ID	Genome size (Mbp)	GC%	Unique ratio	NCBI assembly accession [Assembly version]
*Bacillus subtilis*	bsub	4.1	44.71	0.62	GCF_000009045.1 [ASM904v1]
*Bacillus thuringiensis*	bthu	5.51	36.23	0.44	GCF_000161495.1 [ASM16149v1]
*Bifidobacterium longum*	blon	2.46	60.84	0.53	GCF_004936435.1 [ASM493643v1]
*Chlamydia trachomatis*	ctram	1.04	41.78	0.51	GCF_000008725.1 [ASM872v1]
*Clostridium botulinum*	cbot	3.95	29.48	0.53	GCF_000063585.1 [ASM6358v1]
*Corynebacterium pseudotuberculosis*	cpse	2.32	52.93	0.47	GCF_000144675.2 [ASM14467v2]
*Escherichia coli*	ecol	5.01	51.79	0.62	GCF_000005845.2 [ASM584v2]
*Lactococcus lactis*	llac	2.45	36.86	0.90	GCF_003351805.1 [ASM335180v1]
*Mycobacterium tuberculosis*	mtub	4.4	65.86	0.58	GCF_000195955.2 [ASM19595v2]
*Mycoplasma gallisepticum*	mgal	0.97	32.65	0.89	GCF_000286675.1 [ASM28667v1]
*Pseudomonas aeruginosa*	pae	6.43	66.96	0.32	GCF_000006765.1 [ASM676v1]
*Staphylococcus aureus*	saur	2.82	33.78	0.64	GCF_000013425.1 [ASM1342v1]
*Treponema pallidum*	tpal	1.14	52.6	0.64	GCF_000246755.1 [ASM24675v1]

We selected four sample parameters and for each we specified unique values to be used in the read simulations. Specifically, we used sequencing depth (25 × , 50 × , 75 × , 100 × , 125 × , 150 ×), error rate (0, 0.01, 0.025, 0.05), optical and PCR duplicate ratios (values for both: 0, 0.01, 0.05, 0.15, 0.3). Rationales for the selected values are as follows. Salzberg et al. [23, 24] discusses, among other things, the sequencing depth in a substantial number of studies ranged from 50 × to 100 × . Stoler and Nekrutenko [2] found that the estimated error rate in the middle of the readings is up to about 7%. Accordingly, we assumed a wide range of error rates to cover the wide range of error rates available on different platforms and with different protocols for library preparation. Finally, duplicate rates were found to generally range between 0 and 30% across a wide range of assembly targets [30].

Sequencing libraries were simulated using wgsim 1.10 (https://github.com/lh3/wgsim) as implemented in readSimulator.py 0.0.1 (https://github.com/wanyuac/readSimulator) using 10 iterations to account for the circularity of bacterial chromosomes. Briefly, this script considers a pre-defined sequencing depth and the non-linear topology often observed in prokaryotic genomes, and iteratively generates reads by randomly breaking up the reference genome. We set the mutation rate and fraction of indels to 0 and used the haplotype mode for the simulation of 2 × 150 base pairs paired-end sequencing libraries. We carried out the simulations using all combinations of sequencing depth and sequencing error rate. To introduce optical duplicates, we randomly selected a given fraction of sequencing reads and duplicated the read pairs in the sequencing data. We introduced PCR duplicates similarly, but mutated the randomly chosen read using MutationSimulator 3.0.1 [36] with a SNP-rate of 0.01 before re-introducing the read pair to the dataset. From the described four variables and their values 600 unique combinations could be composed in total. For each unique combination we made 5 independent replications (i.e. sample parameter values were the same, but their realizations were different and independent between iterations), resulting in a nominal sample size of 3000 for each bacterial reference, hence 39,000 simulated libraries for the 13 bacteria in total.

For the de novo assembly of the simulated read data we used SPAdes (ver. 3.13.1; [21]). We chose this specific assembler because currently it is one of the most widely used and robust tools [15]. To faithfully represent the effect of error sources on the assembly, we turned the built-in error correction off (–only-assembler). In other words: even though the general *modus operandi* when using SPAdes is to include the built-in error correction algorithm, we were more curious about the fundamental, unaltered effects of the complex interactions themselves. Also note that even with error correction, some errors are still expected to remain in the sequencing data. However, assessment of (1) how efficient this error correction might be in mitigating the complex effects of the studied sample parameters on assembly quality, or of (2) how efficient different assemblers are in handling such effects, are outside of the scope of this study.

### *De novo* assembly quality metrics

Following the read simulations and assemblies, we assessed the contiguity of the assemblies using QUAST (ver. 5.0.2; [37]) with the corresponding reference genome sequence (i.e. the genome sequence used for the library simulations) specified as reference. For subsequent analyses we used the following metrics (for detailed descriptions please refer to the QUAST manual, with the exception of “GC% bias”, which was calculated by dividing the assembly GC% by the reference GC%):

- total number of contigs,
- number of small contigs,
- number of large contigs,
- size of largest contig (bp),
- N50,
- NG50,
- L50,
- LG50,
- total length of all unaligned regions (bp),
- duplication ratio,
- number of mismatches per 100 kbp,
- number of indels per 100kbp,
- proportion of aligned regions,
- GC% bias.

In the case of the number of small (≤ 200 bp) and large (> 200 bp) contigs, size thresholds were defined as the number of base pairs smaller or equal to, and larger than 200 bp, respectively.

### Statistical analyses

All data handling and statistical data analyses were carried out in R (ver. 4.1.2, [38]).

#### Assembly success

Throughout the assembly attempts (*N* = 39,000) in some cases SPAdes failed to assemble any contigs (see Results: Assembly success), therefore for some sample parameter combinations we have acquired fewer quality metric data points than the nominal sample size. To investigate whether these unsuccessful assemblies occurred at random or not, we fitted a binomial generalized mixed-effects regression model (GLMM) using the R-package “glmmTMB” [39], with the presence of assembly failure as binary response variable (1 = assembly failure occurred), sample parameters as additive continuous predictors, and bacterial reference genome ID as random factor. We also checked whether or not reference genome parameters (genome size, GC%, and unique ratio) affected assembly failures. For this latter we used three Spearman’s rank correlation tests, with each correlating the number of failed assemblies with one of the genome parameters.

#### Quality metrics

To assess the association between sample parameters and de novo assembly quality metrics we utilized a generalized linear regression modeling (GLM) approach, using the quality metrics as response variables, and the sample parameters as predictors. Identification of the adequate model family for parametric models is not always trivial, and after visual inspection of the value distributions of the quality metrics we have found that these distributions varied substantially between metrics, and in some cases even between bacteria for a given metric. Our aims were (1) to achieve the best possible approximation on the effects of sample parameters on quality metrics with reliable fits, and (2) to have estimates of these associations that are comparable across sample parameters and quality metrics as well. In line with these aims we have decided to utilize a flexible and robust two-parameter distribution family, namely the Beta distribution. Beta models were described to handle well numerous probability density function distribution shapes, skewness, and heteroscedasticity [40–42]. Notably, though, Beta models consider a continuous variable with values in the range within the standard limits [0,1], therefore prior to analyses we had to transform quality metrics. We did so by dividing all values of a given quality metric variable by the maximum value, then applying the transformation described in [41]:

$$\begin{array}{c}y=\frac y{y_{max}}\\y''=\frac{y'\left(N-1\right)+1.5}N\end{array}$$

where y represents a quality measure variable, and N is the number of values in the given variable. These transformations were done separately for each bacterial reference. In addition, we also re-scaled the sample parameters using z-score transformation to bring the predictors to the same scale, hence rendering effect size estimates comparable across sample parameters:

$$x'=\frac{x-\mu_x}{\sigma_x}$$

where x represents a sample parameter, µx is the arithmetic mean of x, and σx is the standard deviation of x. Beta GLMs were fitted using the “betareg” R-package [42].

We utilized a two-fold approach throughout modeling. Firstly, we fitted models in which quality metrics were used as response variables and sample parameters as additive continuous predictors. Secondly, we again used quality metrics as responses and sample parameters as continuous predictors, but this time with control for the predictors’ 2-, 3-, and 4-way interactions as well. From here on, we refer to these as additive and multiplicative models, respectively. Our motivation for this two-fold approach was to be able to identify (1) general effects of the assessed sample parameters on the quality metrics and (2) potential interplay between these sample parameters, i.e. their combined (multiplicative) effects on the quality metrics. Practical considerations also played a role: interpretability and post hoc work (see below) with the effect sizes from additive models are quite simpler than marginalizing effect sizes conditioned on other parameter values (e.g. marginal trends for error rate when the other three parameters are at their arithmetic mean of their tested range). In addition, with the same considerations, for every quality metric we fitted separate models for each bacterial reference genome. Therefore we had fitted 182 additive, and 182 multiplicative GLMs (13 bacterial reference genomes, 14 quality metric variables).

After fitting the above described models, we used a meta-analysis approach to robustly assess whether or not given sample parameters have consistent effects on the quality metrics across bacterial genomes, so that we could pool effect sizes and formally test their overall significance. To that end we used the “metafor” R-package [43]. Since all response variables, as well as all predictors were on the same scale, the effect size (and standard error) estimates were used directly in the meta-analytic models, i.e. without prior transformations. In the case of additive models, to estimate pooled effect sizes for the sample parameters on each quality metric we fitted meta-analytic random-effects models, both without and with moderator variables. For the latter, genome size (Mbp), GC%, and unique ratio of the reference bacterial genomes were used. Because during preliminary analyses we’ve observed that in some cases GC% appears to have non-linear associations with some effect sizes, we also included its orthogonal second degree polynomial as moderator. We fitted the meta-regressions separately for moderator variables to avoid multicollinearity between them, which otherwise would have resulted in considerably biased estimates of the meta-regressions.

Marginal trend estimates from models with 3- and 4-way interactions are rather difficult to interpret, especially so in the case of continuous variables. In order to make it a bit more intuitive, it is helpful to select the effect size of a focal predictor, and extract the effect sizes for this focal predictor as a function of the values of the other predictors. When interpreting and presenting our results, we chose to use error rate as the focal predictor, due to its high importance. Consequently, in the case of multiplicative models we extracted the marginal trend estimates (effect sizes) for error rate marginalized to the minimum, average, and maximum values of the other sample parameter values, in all possible value combinations. In other words, we estimated the effect size of error rate for a wide range of scenarios when the other three sample parameters had varying values. For this we used the R-package “emmeans” [44]. Similarly to the meta-analysis of effect sizes from additive models, we applied meta-analytical models both without and with moderator variables (genome size, GC% and [GC%]^2^, and unique ratio of reference genome); in the latter case, we again used separate meta-regressions for each moderator to avoid multicollinearity. Additionally, we pooled effect sizes for the predictor coefficients of the multiplicative GLMs (also with random-effects meta-analytical models), in order to be able to test whether or not interaction terms between the sample parameters consistently influence main effects across reference genomes.

In the Results we mainly report the output of meta-analytical models, presenting the pooled effect sizes of sample parameters on the tested quality metrics. Effect sizes are reported on the logit-scale. Throughout our interpretations we followed the three-sigma rule to identify significant effect size estimates, as we aimed to implement a more conservative threshold on accepting tested effects as significant. In practice this meant that we only considered an effect to be significant with a *P*-value at, or under, 0.003, while we regarded effects with 0.003 < *P* < 0.05 as suggestive (in a similar manner to what was described in [45]). It should be noted that using *P*-value adjustment or estimating false discovery rate (FDR) were not adequate in our analyses, because data are independent across bacteria, whereas the underlying hypothesis is the same. Also note that simple (permutation-based) *P*-value adjustments and FDR estimation don't take context into consideration, whereas pooling effect sizes via meta-analysis implicitly accounts for a context-dependent multiple-test scenario (see Supplementary material for more details).

### Supplementary Information

**Additional file 1.**

## Data Availability

Codes used for the read simulations, assemblies, and quality metric estimations are accessible at the repository of LL (https://github.com/laczkol/readsimulation). The R codes for data handling and analyses, as well as the main data tables can be found at the repository of ZR (https://github.com/zradai/R/tree/master/published_research_analyses/Genome_error_simulations_2022). Generated reads and assemblies are available upon request from ZR.
