# Supplementary material for: An overlooked phenomenon: complex interactions of potential error sources on the quality of bacterial de novo genome assemblies
Source: BMC Genomics. 2024 Jan 9;25:45. doi: 10.1186/s12864-023-09910-4 (PMC10777565; doi:10.1186/s12864-023-09910-4)
Supplement: Supplementary file 1 — Additional file 1. [file 12864_2023_9910_MOESM1_ESM.html]

 

 

 

 
 
 


 An overlooked phenomenon: complex interactions of potential error sources on the quality of bacterial de novo genome assemblies 

 
 
 
 
 
 
 
 
 
 
 
 
 
 
 
 
 
 
 
 
 
 
 
 
 
 
 

 

 
 


 


 

 

 


 


 

 


 


 
 
 
 
 
 

 


 


 An overlooked phenomenon: complex
interactions of potential error sources on the quality of bacterial
 de novo  genome assemblies 
 Supplementary material for review 

 


 
  1  Bacteria 
  
 
 
    Download bacteria 
 
  
 
 
 
  2  Assembly quality metrics 
 
 
 
 
label
 
 
qvar
 
 
 
 
 
 
Duplication ratio
 
 
duplication.ratio
 
 
 
 
GC% bias
 
 
GC.ToRefRatio
 
 
 
 
L50
 
 
L50
 
 
 
 
LG50
 
 
LG50
 
 
 
 
N50
 
 
N50
 
 
 
 
NG50
 
 
NG50
 
 
 
 
Number of indels per 100 kbp
 
 
indels.per.100.kbp
 
 
 
 
Number of large contigs
 
 
contigs.largerThan.200bp
 
 
 
 
Number of mismatches per 100 kbp
 
 
mismatches.per.100.kbp
 
 
 
 
Number of small contigs
 
 
contigs.smallerThan.200bp
 
 
 
 
Proportion of aligned regions
 
 
total.aligned.prop
 
 
 
 
Size of largest contig (bp)
 
 
largest.contig
 
 
 
 
Total length of all unaligned regions (bp)
 
 
unaligned.length
 
 
 
 
Total number of contigs
 
 
contigs.all
 
 
 
 
 
  2.1 
 Total number of contigs  
   
 
 
  2.2 
 Number of small contigs  
   
 
 
  2.3 
 Number of large contigs  
   
 
 
  2.4 
 Size of largest contig (bp)  
   
 
 
  2.5   N50  
   
 
 
  2.6   NG50  
   
 
 
  2.7   L50  
   
 
 
  2.8   LG50  
   
 
 
  2.9 
 Total length of all unaligned regions (bp)  
   
 
 
  2.10 
 Duplication ratio  
   
 
 
  2.11 
 Number of mismatches per 100 kbp  
   
 
 
  2.12 
 Number of indels per 100 kbp  
   
 
 
  2.13 
 Proportion of aligned regions  
   
 
 
  2.14   GC% bias  
   
 
 
 
  3  Sample parameters 
 
 
 
 
label
 
 
evar
 
 
 
 
 
 
Error rate
 
 
err
 
 
 
 
Sequencing depth
 
 
cov
 
 
 
 
PCR duplicate ratio
 
 
pdup
 
 
 
 
Optical duplicate ratio
 
 
odup
 
 
 
 
 
 
  4  Data table 
 
    Download main data table 
 
 
 
  5  Additive models 
 Prior to model fitting, quality metrics were transformed as: 
  \[ y&#39; = \frac{y}{max_y} \] 
 \[ y&#39;&#39; = \frac{y&#39; × (N_{y&#39;} -
1) + 0.5}{N_{y&#39;}} \]  
 where  \(N\)  is the number of
observations. 
 Sample parameters (error rate [err], sequencing depth [cov], PCR
duplicate ratio [pdup], optical duplicate ratio [odup]) were re-scaled
with z-score transformation: 
  \[ x&#39; = \frac{x - \mu_x}{\sigma_x}
\]  
 where  \(\mu_x\)  is the arithmetic
mean of  \(x\) , and  \(\sigma_x\)  is the standard deviation of
 \(x\) . 
 Model formulation: 
  for(b in unique(asq$bact.ID)){
  for(y in qvars){
    
    df.y = na.omit(asq[asq$bact.ID==b,])
    df.y$y = tbeta(df.y[,y]/max(df.y[,y]))
    
    m.y = betareg(y ~ cov.rsc + err.rsc + odup.rsc + pdup.rsc, data = df.y, 
                  control = betareg.control(maxit = 1e4, fsmaxit = 2e3, fstol = 1e-8))
    
  }
}  
 
  5.1  Effect sizes 
 Legend for the figures below: 
 
 dots: regression slope estimates 
 segments: 99.7% confidence interval 
 black dotted vertical line: zero 
 black solid, dashed, or dot-dash vertical line: pooled effect size
based on the simple meta-regression model (no moderators) for
significant (P  \(\le\)  0.003),
suggestive (0.003 &lt; P &lt; 0.05 ) and non-significant (P &gt; 0.05)
effect size, respectively 
 green vertical lane: 99.7% confidence interval of pooled effect size
based on the simple meta-regression model (no moderators) 
 
 Data tables for the estimated marginal trends (EMTs), and
meta-regression (MR) output for MR models without (MR no mods) and with
moderator variables (MR with mods) can be downloaded below: 
 
    Download EMTs 
 
 
    Download MR (no mods) 
 
 
    Download MR (with mods) 
 
 
  5.1.1 
 Total number of contigs  
  ## Warning: Using `size` aesthetic for lines was deprecated in ggplot2 3.4.0.
## ℹ Please use `linewidth` instead.  
   
 
 
  5.1.2 
 Number of small contigs  
   
 
 
  5.1.3 
 Number of large contigs  
   
 
 
  5.1.4 
 Size of largest contig (bp)  
   
 
 
  5.1.5 
 N50  
   
 
 
  5.1.6 
 NG50  
   
 
 
  5.1.7 
 L50  
   
 
 
  5.1.8 
 LG50  
   
 
 
  5.1.9 
 Total length of all unaligned regions (bp)  
   
 
 
  5.1.10 
 Duplication ratio  
   
 
 
  5.1.11 
 Number of mismatches per 100 kbp  
   
 
 
  5.1.12 
 Number of indels per 100 kbp  
   
 
 
  5.1.13 
 Proportion of aligned regions  
   
 
 
  5.1.14 
 GC% bias  
   
 
 
 
  5.2  Genome
parameters 
 
  5.2.1 
 Total number of contigs  
 
  5.2.1.1 
 Error rate  
   
 
 
  5.2.1.2 
 Sequencing depth  
   
 
 
  5.2.1.3 
 PCR duplicate ratio  
   
 
 
  5.2.1.4 
 Optical duplicate ratio  
   
 
 
 
  5.2.2 
 Number of small contigs  
 
  5.2.2.1 
 Error rate  
   
 
 
  5.2.2.2 
 Sequencing depth  
   
 
 
  5.2.2.3 
 PCR duplicate ratio  
   
 
 
  5.2.2.4 
 Optical duplicate ratio  
   
 
 
 
  5.2.3 
 Number of large contigs  
 
  5.2.3.1 
 Error rate  
   
 
 
  5.2.3.2 
 Sequencing depth  
   
 
 
  5.2.3.3 
 PCR duplicate ratio  
   
 
 
  5.2.3.4 
 Optical duplicate ratio  
   
 
 
 
  5.2.4 
 Size of largest contig (bp)  
 
  5.2.4.1 
 Error rate  
   
 
 
  5.2.4.2 
 Sequencing depth  
   
 
 
  5.2.4.3 
 PCR duplicate ratio  
   
 
 
  5.2.4.4 
 Optical duplicate ratio  
   
 
 
 
  5.2.5   N50  
 
  5.2.5.1 
 Error rate  
   
 
 
  5.2.5.2 
 Sequencing depth  
   
 
 
  5.2.5.3 
 PCR duplicate ratio  
   
 
 
  5.2.5.4 
 Optical duplicate ratio  
   
 
 
 
  5.2.6   NG50  
 
  5.2.6.1 
 Error rate  
   
 
 
  5.2.6.2 
 Sequencing depth  
   
 
 
  5.2.6.3 
 PCR duplicate ratio  
   
 
 
  5.2.6.4 
 Optical duplicate ratio  
   
 
 
 
  5.2.7   L50  
 
  5.2.7.1 
 Error rate  
   
 
 
  5.2.7.2 
 Sequencing depth  
   
 
 
  5.2.7.3 
 PCR duplicate ratio  
   
 
 
  5.2.7.4 
 Optical duplicate ratio  
   
 
 
 
  5.2.8   LG50  
 
  5.2.8.1 
 Error rate  
   
 
 
  5.2.8.2 
 Sequencing depth  
   
 
 
  5.2.8.3 
 PCR duplicate ratio  
   
 
 
  5.2.8.4 
 Optical duplicate ratio  
   
 
 
 
  5.2.9 
 Total length of all unaligned regions (bp)  
 
  5.2.9.1 
 Error rate  
   
 
 
  5.2.9.2 
 Sequencing depth  
   
 
 
  5.2.9.3 
 PCR duplicate ratio  
   
 
 
  5.2.9.4 
 Optical duplicate ratio  
   
 
 
 
  5.2.10 
 Duplication ratio  
 
  5.2.10.1 
 Error rate  
   
 
 
  5.2.10.2 
 Sequencing depth  
   
 
 
  5.2.10.3 
 PCR duplicate ratio  
   
 
 
  5.2.10.4 
 Optical duplicate ratio  
   
 
 
 
  5.2.11 
 Number of mismatches per 100 kbp  
 
  5.2.11.1 
 Error rate  
   
 
 
  5.2.11.2 
 Sequencing depth  
   
 
 
  5.2.11.3 
 PCR duplicate ratio  
   
 
 
  5.2.11.4 
 Optical duplicate ratio  
   
 
 
 
  5.2.12 
 Number of indels per 100 kbp  
 
  5.2.12.1 
 Error rate  
   
 
 
  5.2.12.2 
 Sequencing depth  
   
 
 
  5.2.12.3 
 PCR duplicate ratio  
   
 
 
  5.2.12.4 
 Optical duplicate ratio  
   
 
 
 
  5.2.13 
 Proportion of aligned regions  
 
  5.2.13.1 
 Error rate  
   
 
 
  5.2.13.2 
 Sequencing depth  
   
 
 
  5.2.13.3 
 PCR duplicate ratio  
   
 
 
  5.2.13.4 
 Optical duplicate ratio  
   
 
 
 
  5.2.14   GC% bias  
 
  5.2.14.1 
 Error rate  
   
 
 
  5.2.14.2 
 Sequencing depth  
   
 
 
  5.2.14.3 
 PCR duplicate ratio  
   
 
 
  5.2.14.4 
 Optical duplicate ratio  
   
 
 
 
 
  5.3  False discovery rate
(FDR) and Stouffer’s Z-score modification 
 Due to the large total number of hypothesis tests throughout our
analyses, one might prompt that some sort of p-value adjustment and/or
false discovery rate (FDR) estimation is necessary. However, it should
be noted that this would only be strictly true in the case of carrying
out a large number independent hypothesis tests on the same data. In our
case, though, data is independent across bacteria, whereas the
underlying hypothesis is the same. Also note that simple
(permutation-based) p-value adjustments and FDR estimation don’t take
context into consideration. This was one of the main motivations for us
to pool effect sizes in meta-analysis, which implicitly accounts for a
context-dependent multiple-test scenario. 
 The effect size estimates for the different quality metrics assessed
separately per each sample parameter (e.g. effect of error rate on NG50,
effect of sequencing depth on number of contigs), are not part of
independent hypotheses, since every quality metric corresponds to a
relevant aspect of the assemblies’ quality. Therefore when we repeatedly
test the sample parameters’ effects on these, an increased incidence of
significant effects are associated with an increased likelihood that our
findings are genuine, instead of being the result of type I error
(i.e. false positive results). In such a scenario, Fisher’s combined
probability test, as well as Stouffer’s Z-score, give adequate
quantification for the robustness of the results. 
 Fisher’s method includes the estimation of a  \(X^2\)  statistic from the extreme value
probabilities (p-values): 
  \[X^2 = -2 \sum_{i=1}^k log(p_i)
\]  
 where  \(k\)  is the number of
tests. 
 Stouffer’s Z-score is: 
  \[Z = \frac{\sum_{i=1}^k
Z_i}{\sqrt{k}}\]  
 Since we’re interested whether or not a given sample parameter has
consistent effect across quality metrics, we can calculate the above
mentioned quantities separately for each sample parameter. Note that
these methods yield one estimate for each sample parameter, representing
its significance effecting each quality metric. In other words, for each
sample parameter, the number of p-values used in Fisher’s method, and
number of z-scores used in Stouffer’s method, was the number of quality
metrics on which modeling was carried out (n = 14), representing the
significance of the association between that given sample parameter and
quality metrics. FDR may then be applied on the four estimates
(i.e. adjusting the estimates for each sample parameters). 
 Some z-scores were multiplied by  \(-1\)  so that all z-scores all conceptually
consistent with representing a sample parameter’s effect on assembly
quality (i.e. their direction is aligned so negative values represent
adverse, positive values represent advantageous effects): 
 
 
 
 
metric
 
 
multiplier
 
 
 
 
 
 
Total number of contigs
 
 
-1
 
 
 
 
Number of small contigs
 
 
-1
 
 
 
 
Number of large contigs
 
 
-1
 
 
 
 
Size of largest contig (bp)
 
 
1
 
 
 
 
N50
 
 
1
 
 
 
 
NG50
 
 
1
 
 
 
 
L50
 
 
-1
 
 
 
 
LG50
 
 
-1
 
 
 
 
Total length of all unaligned regions (bp)
 
 
-1
 
 
 
 
Duplication ratio
 
 
-1
 
 
 
 
Number of mismatches per 100 kbp
 
 
-1
 
 
 
 
Number of indels per 100 kbp
 
 
-1
 
 
 
 
Proportion of aligned regions
 
 
1
 
 
 
 
GC% bias
 
 
1
 
 
 
 
 On the figure below Fisher’s  \(X^2\) 
and Stouffer’s Z-score are shown for the four sample parameters,
representing the significance of their effects over all quality metrics,
based on their pooled effect sizes from the meta-analysis models. 
  
 
 Estimation of FDR is not possible due to all p-values being zero!
(More precisely: estimated p-values are below the minimum representable
value of R.) 
 
 
 
  6  Multiplicative
models 
 Prior to model fitting, quality metrics were transformed as: 
  \[ y&#39; = \frac{y}{max_y} \]  
  \[ y&#39;&#39; = \frac{y&#39; ×
(N_{y&#39;} - 1) + 0.5}{N_{y&#39;}} \]  
 where  \(N\)  is the number of
observations. 
 Sample parameters (error rate [err], sequencing depth [cov], PCR
duplicate ratio [pdup], optical duplicate ratio [odup]) were re-scaled
with z-score transformation: 
  \[ x&#39; = \frac{x - \mu_x}{\sigma_x}
\]  
 where  \(\mu_x\)  is the arithmetic
mean of  \(x\) , and  \(\sigma_x\)  is the standard deviation of
 \(x\) . 
 Model formulation: 
  for(b in unique(asq$bact.ID)){
  for(y in qvars){
    
    df.y = na.omit(asq[asq$bact.ID==b,])
    df.y$y = tbeta(df.y[,y]/max(df.y[,y]))
    
    m.y = betareg(y ~ cov.rsc * err.rsc * odup.rsc * pdup.rsc, data = df.y, 
                  control = betareg.control(maxit = 1e4, fsmaxit = 2e3, fstol = 1e-8))
    
  }
}  
 
  6.1  Model
coefficients 
 Model coefficients directly from the model outputs: 
 
    Download coefficients 
 
 Coefficients pooled (with meta-regression model without moderator
variables) across bacteria: 
 
    Download pooled coefficients 
 
 
 
  6.2  Effect sizes 
 Data tables for the model coefficients acquired directly from the
model output, estimated marginal trends (EMTs), and meta-regression (MR)
output for MR models without (MR no mods) and with (MR with mods) can be
downloaded below. EMTs were acquired for error rate, marginalized at
different value combinations of the 3 other sample parameters
(sequencing depth, PCR and optical duplicate ratios). 
 
    Download EMTs 
 
 
    Download MR (no mods) 
 
 
    Download MR (with mods) 
 
 
  6.2.1 
 Total number of contigs  
   
 
 
  6.2.2 
 Number of small contigs  
   
 
 
  6.2.3 
 Number of large contigs  
   
 
 
  6.2.4 
 Size of largest contig (bp)  
   
 
 
  6.2.5 
 N50  
   
 
 
  6.2.6 
 NG50  
   
 
 
  6.2.7 
 L50  
   
 
 
  6.2.8 
 LG50  
   
 
 
  6.2.9 
 Total length of all unaligned regions (bp)  
   
 
 
  6.2.10 
 Duplication ratio  
   
 
 
  6.2.11 
 Number of mismatches per 100 kbp  
   
 
 
  6.2.12 
 Number of indels per 100 kbp  
   
 
 
  6.2.13 
 Proportion of aligned regions  
   
 
 
  6.2.14 
 GC% bias  
   
 
 
 
  6.3  Genome
parameters 
 
  6.3.1 
 Total number of contigs  
 
  6.3.1.1 
 size.mb.full  
   
 
 
  6.3.1.2 
 GC  
   
 
 
  6.3.1.3 
 complexity  
   
 
 
 
  6.3.2 
 Number of small contigs  
 
  6.3.2.1 
 size.mb.full  
   
 
 
  6.3.2.2 
 GC  
   
 
 
  6.3.2.3 
 complexity  
   
 
 
 
  6.3.3 
 Number of large contigs  
 
  6.3.3.1 
 size.mb.full  
   
 
 
  6.3.3.2 
 GC  
   
 
 
  6.3.3.3 
 complexity  
   
 
 
 
  6.3.4 
 Size of largest contig (bp)  
 
  6.3.4.1 
 size.mb.full  
   
 
 
  6.3.4.2 
 GC  
   
 
 
  6.3.4.3 
 complexity  
   
 
 
 
  6.3.5   N50  
 
  6.3.5.1 
 size.mb.full  
   
 
 
  6.3.5.2 
 GC  
   
 
 
  6.3.5.3 
 complexity  
   
 
 
 
  6.3.6   NG50  
 
  6.3.6.1 
 size.mb.full  
   
 
 
  6.3.6.2 
 GC  
   
 
 
  6.3.6.3 
 complexity  
   
 
 
 
  6.3.7   L50  
 
  6.3.7.1 
 size.mb.full  
   
 
 
  6.3.7.2 
 GC  
   
 
 
  6.3.7.3 
 complexity  
   
 
 
 
  6.3.8   LG50  
 
  6.3.8.1 
 size.mb.full  
   
 
 
  6.3.8.2 
 GC  
   
 
 
  6.3.8.3 
 complexity  
   
 
 
 
  6.3.9 
 Total length of all unaligned regions (bp)  
 
  6.3.9.1 
 size.mb.full  
   
 
 
  6.3.9.2 
 GC  
   
 
 
  6.3.9.3 
 complexity  
   
 
 
 
  6.3.10 
 Duplication ratio  
 
  6.3.10.1 
 size.mb.full  
   
 
 
  6.3.10.2 
 GC  
   
 
 
  6.3.10.3 
 complexity  
   
 
 
 
  6.3.11 
 Number of mismatches per 100 kbp  
 
  6.3.11.1 
 size.mb.full  
   
 
 
  6.3.11.2 
 GC  
   
 
 
  6.3.11.3 
 complexity  
   
 
 
 
  6.3.12 
 Number of indels per 100 kbp  
 
  6.3.12.1 
 size.mb.full  
   
 
 
  6.3.12.2 
 GC  
   
 
 
  6.3.12.3 
 complexity  
   
 
 
 
  6.3.13 
 Proportion of aligned regions  
 
  6.3.13.1 
 size.mb.full  
   
 
 
  6.3.13.2 
 GC  
   
 
 
  6.3.13.3 
 complexity  
   
 
 
 
  6.3.14   GC% bias  
 
  6.3.14.1 
 size.mb.full  
   
 
 
  6.3.14.2 
 GC  
   
 
 
  6.3.14.3 
 complexity  
   
 
 
 
 
  6.4  False discovery rate
(FDR) and Stouffer’s Z-score modification 
 Same methods as in the Additive models, carried out on the pooled
coefficients of the multiplicative models. 
  
 
 
 
 
 
evar
 
 
stouffer.z
 
 
fisher.chisq
 
 
fisher.p
 
 
local.FDR
 
 
FDR.p
 
 
 
 
 
 
cov.rsc
 
 
-60.123797
 
 
2375.78161
 
 
0.0e+00
 
 
0
 
 
0.0e+00
 
 
 
 
err.rsc
 
 
-84.010427
 
 
3049.49105
 
 
0.0e+00
 
 
0
 
 
0.0e+00
 
 
 
 
odup.rsc
 
 
14.175035
 
 
390.28404
 
 
0.0e+00
 
 
0
 
 
0.0e+00
 
 
 
 
pdup.rsc
 
 
-30.234489
 
 
1688.51652
 
 
0.0e+00
 
 
0
 
 
0.0e+00
 
 
 
 
cov.rsc:err.rsc
 
 
-28.741689
 
 
1673.43311
 
 
0.0e+00
 
 
0
 
 
0.0e+00
 
 
 
 
cov.rsc:odup.rsc
 
 
7.049662
 
 
235.99513
 
 
0.0e+00
 
 
0
 
 
0.0e+00
 
 
 
 
err.rsc:odup.rsc
 
 
10.501991
 
 
282.08246
 
 
0.0e+00
 
 
0
 
 
0.0e+00
 
 
 
 
cov.rsc:pdup.rsc
 
 
13.700289
 
 
720.65975
 
 
0.0e+00
 
 
0
 
 
0.0e+00
 
 
 
 
err.rsc:pdup.rsc
 
 
-28.328542
 
 
1698.38718
 
 
0.0e+00
 
 
0
 
 
0.0e+00
 
 
 
 
odup.rsc:pdup.rsc
 
 
8.917441
 
 
236.36161
 
 
0.0e+00
 
 
0
 
 
0.0e+00
 
 
 
 
cov.rsc:err.rsc:odup.rsc
 
 
10.181845
 
 
220.34234
 
 
0.0e+00
 
 
0
 
 
0.0e+00
 
 
 
 
cov.rsc:err.rsc:pdup.rsc
 
 
24.215382
 
 
813.26193
 
 
0.0e+00
 
 
0
 
 
0.0e+00
 
 
 
 
cov.rsc:odup.rsc:pdup.rsc
 
 
-1.534176
 
 
74.25996
 
 
4.6e-06
 
 
1
 
 
4.6e-06
 
 
 
 
err.rsc:odup.rsc:pdup.rsc
 
 
14.884926
 
 
377.27129
 
 
0.0e+00
 
 
0
 
 
0.0e+00
 
 
 
 
cov.rsc:err.rsc:odup.rsc:pdup.rsc
 
 
-5.953095
 
 
113.94014
 
 
0.0e+00
 
 
1
 
 
0.0e+00
 
 
 
 
  NOTE:   FDR estimates may be unreliable, due to low
number of input statistics (n = 13; censored sample for null model
estimation has only size 1)! 
 
 
 


 
 

 

 

 

 

 

 

 
 

 
 
